# Editorial: Positive Education: Theory, Practice, and Evidence

**DOI:** 10.3389/fpsyg.2020.00427

**Published:** 2020-03-09

**Authors:** Wenjie Duan, Zheng Chen, Samuel M. Y. Ho

**Affiliations:** ^1^School of Social and Public Administration, East China University of Science and Technology, Shanghai, China; ^2^Institute of Education, Wuhan University, Wuhan, China; ^3^Department of Social and Behavioral Sciences, City University of Hong Kong, Hong Kong, China

**Keywords:** positive education, positive psychology, strengths perspective, PERMA-H, well-being

In one or two words, what do we desire most for the young generation? Seligman et al. (2009) once gave a test similar to this question in the beginning of his paper and argued that most people aspire well-being for their children. We believe that “positive education” is another answer to the question.

This paper aims to shed light on pathways and prospects of positive education. In the subsequent pages, we initially review key insights into studies that focused on the psychological well-being of individuals, which provides an empirical foundation for positive education. Moving beyond these studies, we focused on positive education. Studies under this category either adopted a holistic PERMA-H positive education model or examined one or two of its six elements. The PERMA model of flourishing was introduced by Seligman (2011), who classified psychological well-being into five domains, namely, positive emotions (P), engagement (E), relationships (R), meaning (M), and accomplishment (A). In late 2013, another element called positive health (H) was added to the PERMA model to embrace a holistic view of physical and psychological health (Norrish et al., 2013). Finally, we highlight the contributions of this special issue and conclude with suggestions for future study.

## Insights into Individuals' Psychological Well-being

This section addresses the aim of this paper to enhance the young generation's psychological well-being and deal with negative emotions by positive psychology. This group consists of five papers.

Chi et al. evaluated the effects of mindfulness-based stress reduction (MBSR) in the treatment of depression among adolescents and young adults by a systematic review of the literature and metaanalysis. Eighteen randomized controlled trails (RCTs) featuring 2,042 participants are included in the metaanalysis. Results showed that MBSR has modest effects for reducing depressive symptoms post-intervention. The meta-regression suggested that the average treatment effect might be moderated by control condition, treatment duration, and subjects' baseline depression. However, mediators such as individual strengths, timing, and sequence of change (Labelle et al., 2015) and rumination (Labelle et al., 2010), among others are not discussed and further research is required to assess the follow-up effects of MBSR on depressive symptoms. Extremera et al. indicated that students' own ability, particularly emotional intelligence (EI), including self-control skill, plays a buffering role for adolescent victims of cyberbullying from the harm of suicidal ideation and increases their levels of mental health. Thus, EI is an important life skill for adolescent students that should be taught in schools. EI programs may be beneficial to students with test anxiety (TA) problems. Pena and Losada examined this hypothesis and focused on emotional attention and TA. They found that emotional attention is associated with high scores in self-rumination, thereby increasing reliance upon TA. Moreover, self-rumination fully mediates the link between emotional attention and TA. Similar to the notion that TA impairs school students' physical and emotional well-being, competence frustration has been consistently found to undermine one's intrinsic motivation in the same activity. Fang et al. explored a single basic psychological need (competence frustration) involving a sample of 617 undergraduate students in China and found an interesting phenomenon that was previously unreported. Notably, if competence frustration in this activity is sufficiently high and exceeds a critical point, then a restoration process would be activated to help individuals regain competence in the subsequent less-demanding activity. Thus, participants would exhibit enhanced intrinsic motivation toward the subsequent activity. Puente-Martínez et al. provided a reliable and valid measure of negative affect regulation to relieve negative emotions by involving a sample of 264 university students. They argued that negative affect regulation has greater relevance with eudemonic than hedonic well-being. The results of this study were beneficial for promoting emotional capacities to effectively cope with negative situations in educational contexts.

These five aforementioned papers provide certain empirical evidence that helps us understand how individuals, from toddler through adolescent stages, develop resiliency amidst difficulties and maladaptation. That is, their inner power or emotional ability helps overcome negative emotions and protect their mental health and well-being.

## Approaches to Positive School Education

Several group of papers are included for satisfying the criterion of focusing on the flourishing of young people, which is currently a well-established and measurable psychological construct worldwide (Silva and Caetano, 2011; Sumi, 2013; Villieux et al., 2016; Duan and Xie, 2019). The two papers adopted a holistic PERMA or PERMA-H positive education model (Norrish et al., 2013) whereas the eight remaining papers focused on one or two elements of positive education (Seligman, 2011). The six elements include positive emotions, positive engagement, positive relationships, positive purpose (meaning), positive accomplishment, and positive health that is underpinned by focusing on character strengths.

Two papers (Shoshani and Slone; Lai et al.) contributed to the PERMA-H model through large-scale intervention programs in schools. Shoshani and Slone took preschool students as subjects and investigated the effects of positive psychology interventions on their subjective well-being, mental health, and learning behaviors through a RCT during one school year. The program focused on the four elements of the PERMA model including activities for enhancement of positive emotions, engagement, relationships, and achievement. Their findings revealed significant increases in subjective well-being and positive learning behaviors in the intervention group, whereas no significant changes were found in the control group. Thus, the potential of positive psychological interventions for increasing subject well-being and positive learning behaviors at young ages was highlighted. The results were consistent with the conclusion of an early review on positive psychology interventions (PPI) in 12 schools from kindergarten to high school and PPI RCTs in other stages of life such as freshmen and adults (Gander et al., 2016). The ability of PPI to build the well-being of students have been manifested overtime and across cultures. Lai et al. validated a multidimensional measure of PERMA-H in the context of a positive education program evaluation among senior primary school students to provide a solid foundation for related scientific research and the understanding of the multidimensional framework of positive psychology concepts. The set of measurements would be an invaluable tool for the development of positive education in Hong Kong or other parts of China.

The eight remaining papers focused on a part of the positive education model. Research has shown that character strengths are linked to positive youth development (Park et al., 2004; Duan et al., 2015) and numerous valued outcomes (e.g., decreases in depression, increase in life satisfaction, and achievement) (Duan and Bu, 2017; Schutte and Malouff, 2019). In line with previous research, Zhang and Chen discovered that certain character strengths, namely, hope, curiosity, zest, perseverance, and love bear the strongest correlations with subjective well-being. Future self-continuity moderates the mediation of strength use because it moderates the effect of strength use on subjective well-being. These findings expand knowledge on future self-continuity and its relation to strength use and subjective well-being among undergraduates. Zhao et al. focused on growth mindset and grit and examined the mediating roles of leaning motivations. Results from a cross-sectional investigation administered to 1,842 students in a Chinese city indicated that learning motivations partially mediate the relationship between growth mindset and grit. This study extended the understanding of the underlying mechanisms through which growth mindset and grit positively influence education. Li et al. unveiled that students' previous achievements predicted their behavioral, emotional, and cognitive engagements, and the association between previous achievement and school engagement is strong among students under the scenario that incorporates incremental theory but not for those under the scenario that incorporates entity theory in emotional and cognitive engagements. Their findings revealed that students with positive notions of intelligence may perform better than those with negative notions. Drawing upon self-determination theory and previous studies, Orkibi and Ronen hypothesized that the association between self-control skills (SCS) and their subjective well-being may be mediated by students' perceived satisfaction of their basic psychological needs for competence, relatedness, and autonomy. The findings support the hypothesized model. Self-control skills are actual predictors of perceived needs satisfaction and, consequently, subjective well-being (SWB) SWB in school. Yu et al. attempted to understand how academic achievement is affected by subjective well-being. Therefore, they investigated 434 university students in Hong Kong through an online survey. The results showed that personal well-being exhibits long-standing positive effects on university engagement and thus on learning achievement during university years. Therefore, the promotion of holistic development among university students should be given increased attention. Widlund et al. examined a number of academic well-being (e.g., school burnout, schoolwork engagement, and mathematics self-concept) and mathematics performance profiles, apart from subjective well-being. These profiles were relatively stable across one school year during seventh to ninth grades. In addition, academic well-being and mathematics are positively related to students' educational aspirations. Steinmayr et al. investigated school-related and individual predictors of subjective well-being and academic achievements. Positive school climate including self-efficacy and the worry component of TA predicted SWB and/or GPA. Given that the family is also a key resource for children to grow and thrive, Duan et al. utilized the data from 19,487 Chinese junior school students from the 2013–2014 Chinese Educational Panel Survey and analyzed the effects of parental involvement and socioeconomic status on their children's academic achievements and school behaviors. The findings implied that parental involvement activities are highly beneficial for junior school students in families with low socio-economic status (SES). Academic socialization is generally associated with academic success, whereas home-based involvement closely relates to school behaviors. Thus, the promotion of parental involvement in low SES families should be given increased attention.

Together, these eight papers clarify a multidimensional positive education model by explaining how to understand, implement, and evaluate the model.

## Future Horizons for Positive Education Research

This special issue aims to propose a leading-edge research from positive psychology and positive education literature to enhance our understanding of well-being and its significance in the field of education in this era that focuses on well-being as the ultimate goal of human societies. We begin by outlining five papers in the field of positive psychology, which determine the underlying mechanisms of young people's mental health and its relations to their academic achievements. Subsequently, we introduce 10 papers that are built on the concept of positive education or related to elements of positive education.

This introductory article aims to highlight the studies in this special issue and motivate readers to appreciate and build on these contributions. White (2016) suggested that we must provide scientific evidence, organizational benefits, and philosophical arguments to support the integration of well-being in education. Therefore, we intend to provide suggestions following White's opinion.

First, robust evidence-based and scientifically informed results should be provided to researchers and policymakers to emphasize that well-being matters in its own right. Thus, enhanced and well-controlled tests, diachronic studies, large-scale investigations, cross-cultural studies, and metaanalysis should be conducted.

Second, better compelling cases of intervention programs than those previously conducted should be established to demonstrate the benefits of positive education and psychologically informed approaches to educational leaders, school educators, parents, and other shareholders. This approach may involve schools at all levels and of various types, from kindergartens to universities and from high-performing students to students with learning difficulties.

Third, positive parenting (Seligman, 2002) and its relationship to students' well-being and performances should be valued. To promote the research in this cross-context field, additional evidence is required through systemic investigations and interventions (Sanders et al., 2014). The increased understanding on the influence of families on students' well-being and school performances further bonds the ties that we build for schools and families. Thus, the vision of positive education will be clear for the young generation.

## Author Contributions

ZC wrote the draft. SH revised and commented on the previous submission. WD finalized the version and submitted it to the journal.

### Conflict of Interest

The authors declare that the research was conducted in the absence of any commercial or financial relationships that could be construed as a potential conflict of interest.
